# Black holes in bone – irresistible attractors of foreign materials?

**DOI:** 10.1080/17453670902804489

**Published:** 2009-02-01

**Authors:** Per Aspenberg

**Affiliations:** per.aspenberg@inr.liu.se

Bone has a mechanical function. A material to replace it should therefore be able to take over this function. Screws and plates do this, so literally speaking steel is really a bone replacement material. But this term is usually reserved for other materials: various calcium phosphates, calcium sulphate (actually plaster of Paris), bioglass, polymers, titanium granules, chitosan etc. It is thought that a perfect bone replacement material should be biocompatible and have good mechanical strength, but should also (for some reason) be replaced by host bone over time. In addition, it should preferably be injectable.

Replacement of the implanted material by host bone is a complex procedure. It requires that the material should be porous and have an “osteoconductive” surface, but it must also have the ability to dissolve or be resorbed by osteoclasts. Dissolution is virtually impossible, however, once the material has fulfilled its function and become covered and sealed by host bone. If it is dissolved before being covered, it cannot, by definition, be osteoconductive.

When, then, does bone need to be replaced? By far the most common indication is bone loss due to prosthetic loosening. Here, we mainly use compacted allogeneic bone grafts. This material contains immunogenic tissue components, remnants of cartilage, fibrous tissue, and bone marrow, all necrotic. Its osteoconductive or osteoinductive properties are weak or absent. Still, it works marvellously well! It takes up load in a suitably elastic way, is quickly infiltrated by fibrous tissue, and slowly becomes replaced by host bone (which might not be necessary for its function). Considering the composition of allogeneic bone grafts, it is tempting to think that other materials with similar mechanics might be even better. But who dares to change a winning team?

Intercalary bone defects are too large to be treated with bone replacement materials: osteoconduction is a matter of millimeters, not centimeters. So, excluding prosthesis revision and large defects: which indications remain for these materials? – Bone cysts! Bone cysts have aroused (false) hopes for many biomaterial researchers and companies who have a new material, searching for an indication. In this issue of Acta Orthopaedica, Hirn et al. (pp 4-8) and Yanagawa et al. (pp 9-13) demonstrate that benign bone cysts need no other treatment than curettage. Moreover, if fracture still occurs, this is during the first postoperative period, before any bone replacement material would have been incorporated and added to bone strength. This is what many orthopedic surgeons already believe, based on experience. It is good to have evidence now. Much unnecessary grafting can now be avoided. But will it?

**Figure:**
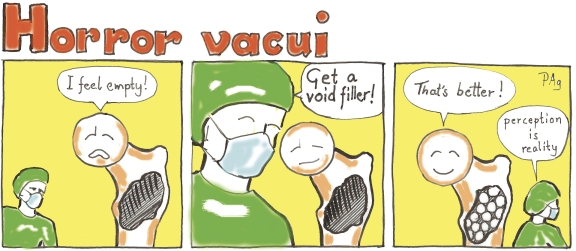


It is likely that the radiographic appearance of voids in the bone steer the thoughts of action-inclined observers, who think that an abnormality in itself is a call for correction. This is really fear of emptiness (*horror vacui*), transformed into a surgical indication. (Actually, *horror vacui* is not a psychological term, but belongs to Aristotelean physics). The same psychology probably lies behind the idea that bone replacement materials should be resorbable: when the void is filled with a material, radiographs still look abnormal. We forget that patients don't care about how their radiographs appear—but about their interpretation, for which we are responsible. Let's hope that the papers by Hirn et al. and Yanagawa et al. get all the attention they deserve. As Göran Bauer, previous Editor in Chief of Acta Orthopaedica, often said: “Retreat from overtreatment”!
